# Inverse Design of Optical Color Routers with Improved Fabrication Compatibility

**DOI:** 10.3390/nano16040251

**Published:** 2026-02-14

**Authors:** Sushmit Hossain, Zerui Liu, Nishat Tasnim Hiramony, Tinghao Hsu, Himaddri Roy, Hongming Zhang, Wei Wu

**Affiliations:** Ming Hsieh Department of Electrical and Computer Engineering, University of Southern California, Los Angeles, CA 90089, USA; hossains@usc.edu (S.H.); zeruiliu@usc.edu (Z.L.); hiramony@usc.edu (N.T.H.); tinghao@usc.edu (T.H.); himaddri@usc.edu (H.R.); hongming@usc.edu (H.Z.)

**Keywords:** inverse design, color router, CMOS image sensors, reflective display, color filter, metasurface

## Abstract

We present a Genetic Algorithm (GA)-based inverse design framework for creating a single-layer, fabrication-compatible dielectric nano-patterned surface that enables efficient color routing in both transmissive and reflective optical systems. Unlike traditional multilayer or absorption-based color filters, the proposed structure employs a fabrication-compatible architecture that spatially routes red, green, and blue light into designated output channels, significantly enhancing light utilization and color fidelity. The design process integrates a GA with full-wave finite-difference time-domain (FDTD) simulations to optimize the structural pillar height distribution, using a figure of merit that simultaneously maximizes optical efficiency and minimizes spectral crosstalk. For CMOS image sensor-scale designs, the nano-patterned surface achieved peak optical efficiencies of 76%, 72%, and 78% for blue, green, and red channels, respectively, with an average efficiency of 75.5%. Parametric studies further revealed the dependence of performance on pillar geometry, refractive index, and unit cell scaling, providing practical design insights for scalable fabrication using nanoimprint or grayscale lithography. Extending the approach to reflective displays, we demonstrate tunable-mirror-based architectures that emulate electrophoretic microcapsules, achieving efficient color reflection and an expanded color gamut beyond the sRGB standard. This single-layer, inverse-designed nano-patterned surface offers a high-performance and fabrication-ready solution for compact, energy-efficient imaging and display technologies.

## 1. Introduction

Sub-wavelength optical structures have emerged as a pivotal area in contemporary photonics research, drawing intense interest because they enable sub-wavelength control over the fundamental properties of light: amplitude, phase, and polarization. These devices are classified as planar nanostructured optics, typically composed of periodic or aperiodic arrays of sub-wavelength scattering elements, often referred to as meta-atoms [1,2,3,4,5]. These structures allow for significantly finer control over light manipulation, whereas refractive optics are traditionally reserved for controlling light in macroscopic or larger-scale systems.

The underlying principle lies in the ability to locally tailor the electromagnetic boundary conditions across the surface. This allows designers to impose arbitrary and precise changes to the wavefront, including phase shifts, amplitude attenuation, or polarization conversion, all within a device thickness that is generally a fraction of the operating wavelength [6,7]. This unprecedented control over light within a minimal footprint is the key to realizing compact, multifunctional optical elements—including high-numerical-aperture metalenses [8], complex beam-shapers [9], advanced holograms [10], and sophisticated polarization multiplexers [11]. This miniaturization potential holds great promise for next-generation systems in fields ranging from consumer electronics and remote sensing to augmented reality and high-speed communications.

However, the realization of optimal sub-wavelength structure design is fundamentally hampered by the conventional forward-design approach. In this traditional workflow, a designer must manually select candidate meta-atom shapes (e.g., circular posts, elliptical cylinders, complex free-form shapes) and discrete dimensions (size, orientation, period, height, and material composition). They then simulate the optical response using computationally intensive full-wave electromagnetic solvers, sweep parameters, and iteratively optimize the structure. Because the design parameter space is vast (covering geometrical parameters and material selection) and the resulting optical responses are highly nonlinear, many designs require repeated, lengthy rounds of trial and error and significant human intuition. Consequently, the design turnaround time becomes a severe bottleneck. To overcome the inherent inefficiencies and limitations of the forward-design paradigm, recent research has increasingly turned to inverse-design methodologies [12,13]. The core innovation of the inverse-design framework is the reversal of the traditional design flow. Instead of starting with an initial geometry and simulating its outcome, the process begins with the desired optical outcome, such as a target phase map, a specific diffraction pattern, or a spectral splitting behavior and employs sophisticated optimization algorithms to derive the meta-structure geometry that yields that target outcome. Inverse design relies on diverse optimization algorithms, including gradient-based adjoint methods, topology optimization, genetic algorithms, and machine-learning-based generative models [14,15,16,17]. This automated reversal of the design process often leads to highly efficient, non-intuitive geometries that can significantly outperform structures based on human intuition. For example, inverse design has successfully produced metasurfaces with record-breaking efficiencies for aberration-corrected metalenses [18], beam splitters [19], and a highly efficient broadband reflector [20], among a multitude of other applications. The power of this approach lies in its ability to rapidly explore a massive parameter space, accelerating the discovery of high-performance architectures.

**Figure 1:**
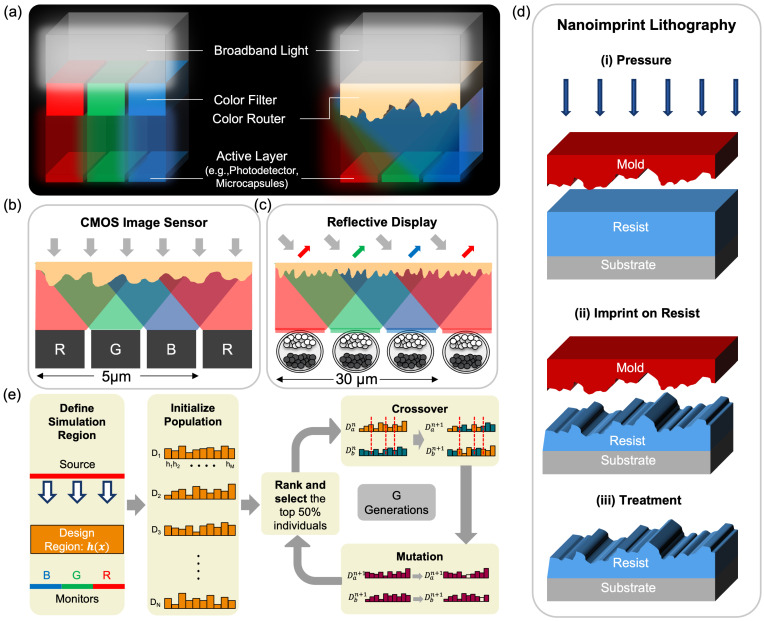
(**a**) Conceptual comparison between traditional color-filter-based systems and our single-layer, height-varying color-routing surface, which directs broadband light into different spectral channels before reaching the active layer (e.g., photodetectors or microcapsules). (**b**) Implementation of the color router in CMOS image sensors, where routed R/G/B components are delivered to spatially separated photodiodes within a compact 5 µm pixel pitch. (**c**) Use of the same color routing concept in reflective displays, where routed colors illuminate different microcapsule regions to modulate reflected light. (**d**) Nanoimprint lithography workflow used to fabricate the nano-patterned surface: (**i**) mold pressing onto the resist, (**ii**) pattern transfer by imprinting, and (**iii**) post-imprint treatment to finalize the nanoscale topography. (**e**) Overview of the genetic-algorithm-based inverse-design framework used to generate manufacturable color-routing structures, including initialization, ranking, crossover, and mutation over multiple generations. The red dashed lines represent crossover segmentation.

Among the diverse applications benefitting from metasurface innovation, color manipulation stands out as a critical domain for commercial impact. Conventional color systems—such as color-filter (CF) arrays used in nearly all CMOS image sensors and display systems suffer from severe optical inefficiency [21,22]. In these architectures, color selection relies on absorption: a CF array transmits only one primary color (red, green, or blue) while blocking or absorbing the other two. Consequently, only about one-third of the incident light is utilized even under ideal conditions, while additional losses from reflection, scattering, and metal interconnects further reduce efficiency. Color routers (CRs) provide a transformative alternative by replacing absorption-based filtering with wavelength-dependent spatial routing. Instead of discarding unwanted spectral components, a CR directs each color channel toward its designated sub-pixel or detector [23]. This routing mechanism enables far higher light utilization. Figure 1a compares the CF- and CR-based architectures: while CFs pass only one-third of the incoming light, CRs redirect light to its proper sub-pixel without absorption loss.

For CMOS image sensors (CISs), CRs address the photon collection problem caused by shrinking pixels. By routing more photons to the photodiodes, CRs fundamentally improve low-light imaging, increase the signal-to-noise ratio (SNR), and enhance dynamic range. For reflective displays (like electronic paper [24]), CRs can maximize the utilization of ambient light. This replacement of absorption-based filtering boosts effective luminance and color purity, leading to brighter displays with lower power consumption. Figure 1b,c show that this concept scales from typical CIS pixel sizes (5 μm) to reflective display pixels (30 μm or larger).

Several studies have investigated color routers (CRs) for CMOS image sensors [25,26,27,28,29,30,31]. Most employ freeform designs that achieve high optical efficiency [25,26,27], but these architectures are difficult to fabricate due to multilayer complexity, stringent alignment tolerances, and the need for nonphysical refractive index values between air and the metasurface material. Recent efforts toward single-layer, fixed-height metasurfaces [28,29,30,31] have improved manufacturability but at the cost of reduced efficiency, limited by fewer design degrees of freedom. To overcome these challenges, we propose a height-varying single-layer color router that combines high optical performance with fabrication compatibility. The added height modulation provides greater control over optical phase and resonance without requiring ultra-fine lateral features. Our design is compatible with scalable, high-throughput fabrication methods such as nanoimprint (Figure 1d) and grayscale lithography [32,33,34]. In the nanoimprint lithography process, the patterned mold (the mold can be created with the help of greyscale lithography) is pressed onto a polymer resist layer coated on the substrate under applied pressure. The mold displaces the resist, forcing it to conform to the nanoscale features on the mold surface and forming a negative replica of the mold pattern. After imprinting, the resist is solidified through thermal or UV treatment, and the mold is removed to leave behind the patterned resist profile on the substrate, ready for subsequent processing steps. This fabrication process enables the practical implementation of our nano-patterned surface in CMOS image sensors and reflective display systems.

This work employs a Genetic Algorithm (GA)-based inverse design approach to develop a single-layer manufacturable nano-patterned structure for efficient color routing. Section 2 outlines the inverse design methodology and optimization process, emphasizing how the GA explores a wide solution space to overcome the local-minima limitations of gradient-based methods. Section 3 presents the optical performance of the color router, including simulated intensity distributions and color gamut analysis, which are critical for evaluating practical imaging and display performance. We also conduct parametric studies to identify key geometric and material dependencies, providing design insights relevant to large-scale fabrication. Building on these results, the design methodology is further applied to a larger color router unit cell tailored for reflective display applications. The resulting device demonstrates wavelength-selective reflection and enhanced light utilization compared to conventional color-filter-based architectures. Overall, this height-varying, GA-optimized design offers a manufacturable and high-efficiency solution for next-generation color routing technologies in both imaging and reflective display systems.

## 2. Materials and Methods

### Genetic Algorithm-Based Inverse Design

Genetic algorithm [35] provides clear advantages over gradient-based methods for this problem. Gradient-based approaches [36] are fast but naturally produce smoothly varying refractive-index profiles, which are not directly manufacturable; even with binarization steps [37], they remain sensitive to initialization and easily converge to local minima in a highly nonconvex landscape. In contrast, genetic algorithms work with discrete design parameters and explore the search space globally, allowing them to avoid local minima and directly produce fabrication-ready structures. The overview of our Genetic Algorithm (GA)-based inverse design framework is illustrated in Figure 1e. The process begins by defining the simulation region, which employs Perfectly Matched Layers (PMLs) along the y-direction and periodic boundary conditions along the x-direction. Within this region, the design domain is parameterized as a one-dimensional array of pillar heights, where each pillar has a fixed width but a variable height. To avoid deep trenches and maintain structural integrity, the pillars are placed adjacently without any spacing between them. Thus, the surface can be represented as a continuous 1D height profile along the x-direction, denoted by h(x).

During the simulation, a broadband source centered at a wavelength of 550 nm is used. As light propagates through the nano-patterned surface, three virtual monitor regions are defined, corresponding to the active layers of specific device architectures (e.g., photodetectors in CMOS image sensors or reflective layers in display applications). The transmitted optical power at three representative wavelengths (i.e., 450 nm (blue), 550 nm (green), and 650 nm (red)) is recorded at these monitor regions. These transmission data are used to compute the figure of merit (FOM) that drives the optimization process.

The FOM simultaneously maximizes the *optical efficiency (OE)* and minimizes the *optical crosstalk (OX)*. The optical efficiency of a color channel *i*, (oe(i)) is defined as the ratio of the transmitted power of the *i*-th color at its corresponding channel ($P_{i,i}$) to the total incident power of that color ($P_{i,0}$). Conversely, the optical crosstalk for color *i* at channel *j* (ox(i,j)) quantifies the leakage of the *i*-th color into the *j*-th channel, normalized to the input power of color *i*. Mathematically, these quantities and the FOM are expressed as follows:

(1) FOM=αOE−(1−α)OX

(2) $$OE=\sum_{i=R,G,B}oe\left(i\right)=\sum_{i=R,G,B}\frac{P_{i,i}}{P_{i,0}}$$

(3) $$OX=\sum_{i\neq j}ox\left(i,j\right)=\sum_{i\neq j}\frac{P_{i,j}}{P_{i,0}}$$

In all simulations, we set the weighting factor α=0.9, emphasizing the optimization of optical efficiency while maintaining low crosstalk. The selection of this cost function was motivated by multiple previous research articles [25,26,27], which have shown high optical efficiencies and low crosstalk due to the formulation. An alternate way to implement the cost function is to use a gaussian kernel for the transmittance spectra [30]. However, we have chosen this cost function to penalize optical crosstalk in our optimization. A comprehensive comparison with some of the state-of-the-art color routers will be shown later in the article.

Following the definition of the simulation domain and the FOM, the GA-based inverse design is initiated with a population of *N* randomly generated height distributions, referred to as *individuals*. Each individual represents a candidate design, composed of multiple *genes*, where each gene corresponds to the height of a single pillar. The entire population of *N* individuals is evaluated through finite-difference time-domain (FDTD) simulations using MEEP (v1.31.0) [38] to determine their fitness values, defined by the computed FOM.

After evaluation, individuals are ranked according to their fitness, and the top N/2 are selected as parents for the *crossover* stage. During crossover, pairs of parent designs are chosen randomly, and their genes are recombined at random crossover points to produce two offspring. This process continues until a new generation of *N* offspring is produced. Each offspring then undergoes a *mutation* step, where one randomly selected gene is replaced with a random value within a predefined range. The probabilities of crossover and mutation are set to 80% and 20%, respectively.

The newly generated offspring population is then evaluated and ranked again, completing one generation of the optimization loop. This evolutionary process continues for *G* generations. The crossover and selection stages drive the population toward higher overall fitness (i.e., improved FOM), while the mutation stage introduces diversity, helping the algorithm escape local optima and converge toward a global solution.

## 3. Results and Discussion

### 3.1. Optical Performance of the Color Router

**Figure 2:**
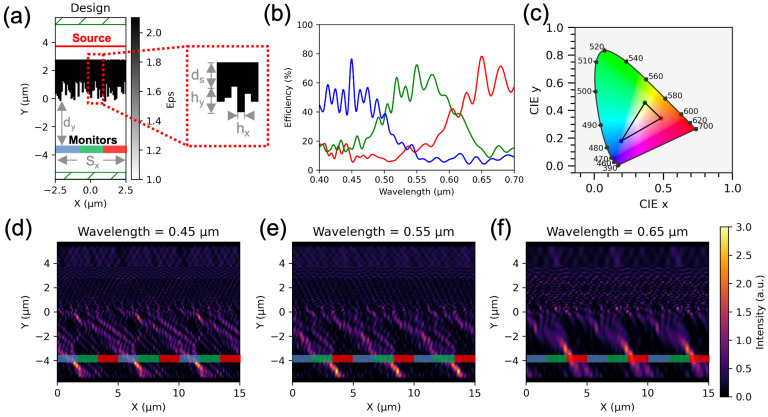
Optical performance of the inverse-designed color router. (**a**) Permittivity map of the optimized structure with highlighted geometric parameters along with the simulation layout showing the incident source and collection monitors. (**b**) Simulated optical-efficiency spectra, demonstrating selective routing of blue, green, and red wavelengths into their respective output channels. (**c**) Corresponding color gamut of the routed outputs plotted in the CIE 1931 chromaticity diagram. (**d**–**f**) Electric-field intensity maps showing wavelength-dependent routing behavior for (**d**) blue light (450 nm), (**e**) green light (550 nm), and (**f**) red light (650 nm), confirming efficient spatial separation of the spectral bands.

Figure 2a illustrates the nano-patterned color router designed through the Genetic Algorithm (GA)-based inverse design process. In this particular case, the structure was implemented with a substrate thickness of $d_{s}=1.5$ μm, a maximum pillar height of $h_{y}=1.5$ μm, a pillar width of $h_{x}=100\;\mathrm{nm}$, a color router to monitor separation distance of $d_{y}=3.5$ μm, and a unit cell width of $S_{x}=5$ μm. After running the inverse design algorithm with a population size of 50 for 500 generations, the optimized structure shown in Figure 2a was obtained.

The broadband transmission characteristics of this optimized surface are presented in Figure 2b. The spectra display the transmission efficiencies measured at the three virtual monitor positions. Here, the efficiency is defined as the ratio of the optical power collected at a specific monitor to the total input optical power incident on the system. As observed from the results, the blue, green, and red monitor locations exhibit peak optical efficiencies of 76%, 72%, and 78%, respectively, corresponding to an overall mean efficiency of 75.5%. These results confirm that the color router effectively separates the incoming broadband light into the desired color channels with high efficiency. We have discussed fabrication tolerance, polarization sensitivity and oblique incidence sensitivity in Sections S1 and S2 of the Supplementary Document.

Based on the obtained efficiency spectra, the achievable color gamut is plotted in Figure 2c. This diagram represents the range of colors that can be reproduced solely by the color router, without the inclusion of external enhancements such as additional color filters or multilayer coatings. Further improvements to this color gamut will be discussed later in the article.

Figure 2d–e present the single-wavelength optical responses of the color router. These plots show the spatial intensity distributions when the device is illuminated with monochromatic light at 450 nm (blue), 550 nm (green), and 650 nm (red), respectively. For clarity, three unit cells are shown in each case to illustrate the field propagation and interaction across the color router. The intensity maps clearly demonstrate that each wavelength is efficiently directed toward its designated output channel, validating the color-routing functionality achieved through the GA-based design process. We also provide phase distribution diagram in Supplementary Section S3 to discuss potential underlying mechanism.

The spectral routing performance of the height-varying structure is inherently linked to the filter characteristics of the target primary colors. Mathematically and physically, the blue and red routing channels function as low-pass and high-pass filters, respectively, whereas the green channel requires a more complex bandpass response. From a design and optimization standpoint, high- or low-pass transitions are significantly less complex to engineer, as they necessitate precise phase control at only a single spectral boundary. Consequently, the increased difficulty in defining two sharp spectral transitions for the green bandpass channel leads to observable crosstalk at the blue-green (B-G) and green-red (G-R) interfaces.

**Figure 3:**
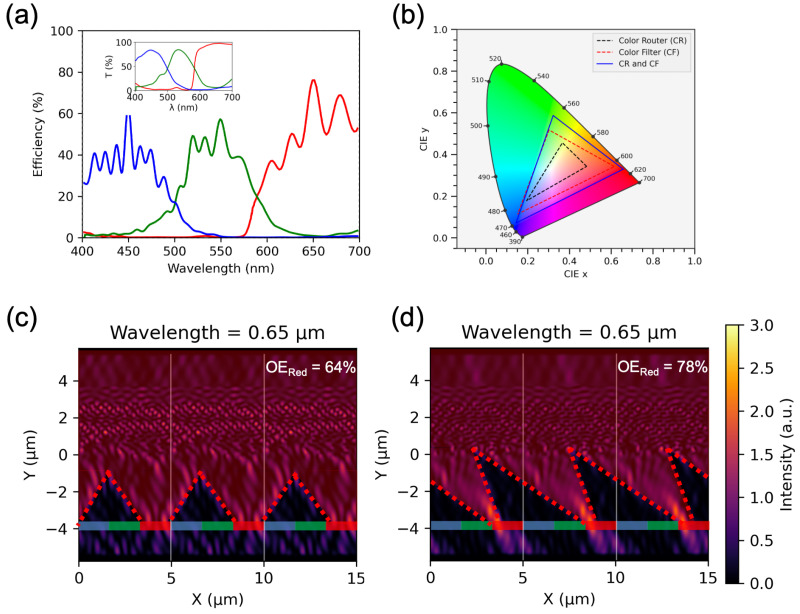
(**a**) Efficiency spectra of a CR-CF hybrid system. Inset shows wavelength dependent transmittance of a commercial color filter. (**b**) Color reproduction comparison of CR, CF and CR-CF hybrid systems (**c**) Intensity distribution for an optimized structure with lower optical efficiency for red color. (**d**) Intensity distribution for an alternate optimized structure with a higher optical efficiency for red color, illustrating the effect of additional iterations during optimization.

Crosstalk in the CR efficiency spectra can further be reduced using a CR-CF hybrid system, as shown in Figure 3a. Here we combine our color router response with a commercially available color filter [39]. The transmittance of the color filter is shown in the inset of Figure 3a. The hybrid system is able to reduce optical crosstalks which results in a larger gamut than CR or CF implemented individually(Figure 3b). Due to the insertion of the CF into the system, the optical efficiency faces a slight decrease. The efficiency of the hybrid system reduces to 64%, 57% and 76% for blue, green and red, respectively. This results in a mean optical efficiency of 65.67%, which is also 99% higher than a standard CF-based system.

Figure 3c,d compare two distinct outcomes obtained from the inverse design process. These figures display the optical intensity distributions for two structures under normal incidence of red light (650 nm). The design shown in Figure 3c corresponds to an optimization run with fewer generations, whereas the structure in Figure 3d was obtained after a larger number of generations, allowing the algorithm to find a better optima.

From a conventional design perspective, one might intuitively expect the optimized structure to exhibit a mode profile resembling that of a metalens, as observed in Figure 3c. However, despite its visually familiar appearance, this configuration represents a local minimum in the optimization landscape rather than the global optimum. The corresponding optical efficiency values confirm that it is not the most effective solution.

When the Genetic Algorithm was allowed to run for more generations, the optimizer identified a different structure with an unconventional yet more efficient field distribution, as illustrated in Figure 3d. This outcome reflects the algorithm’s ability to escape local minima and converge toward a superior solution. Although the resulting intensity profile deviates from traditional lens-like behavior, it achieves higher optical efficiency for the red channel and preserves the structure’s intended color-routing functionality. This comparison highlights the advantage of extended optimization in discovering non-intuitive but high-performing photonic designs.

### 3.2. Parametric Analysis

**Figure 4:**
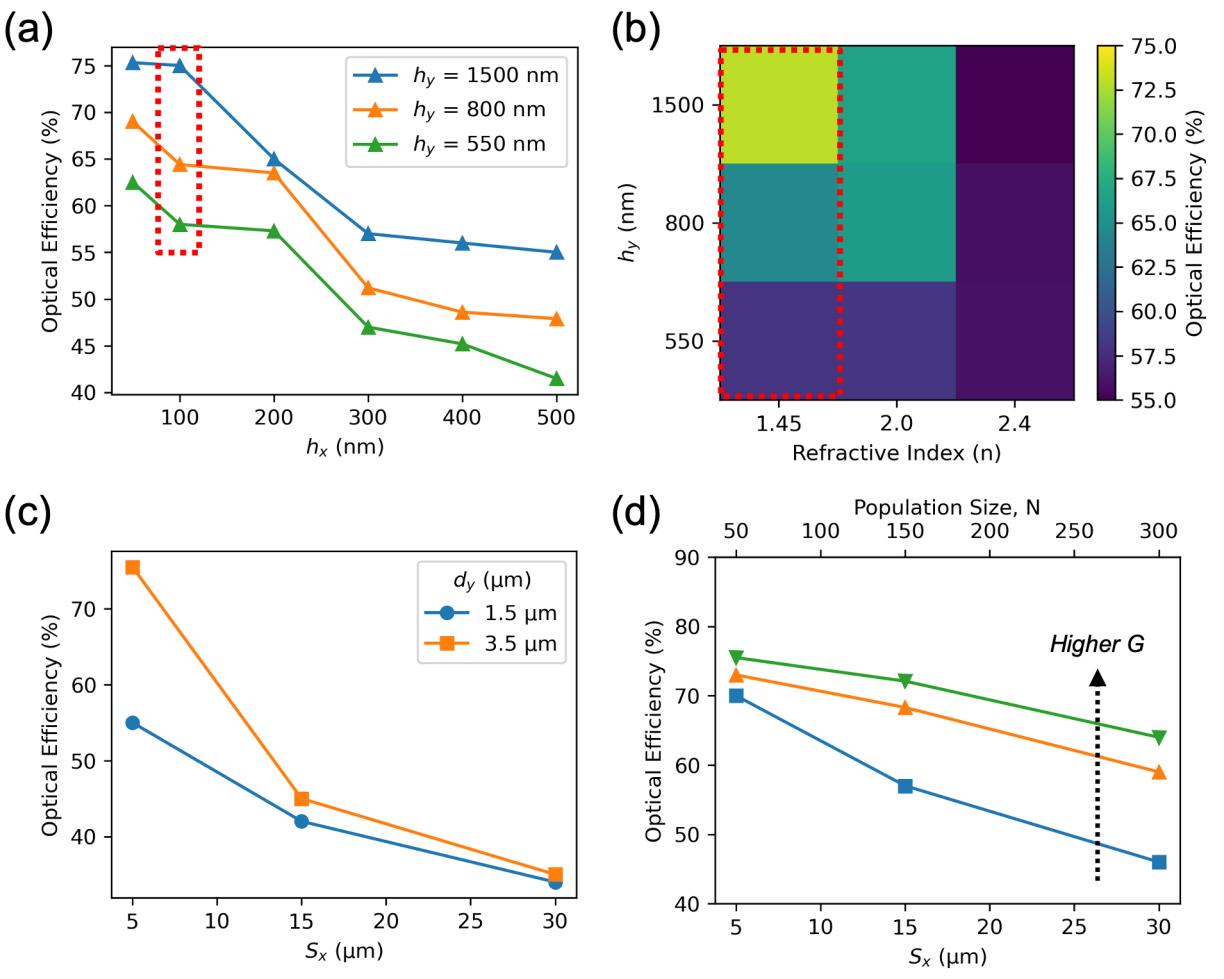
Parametric analysis of color router performance. (**a**) Optical efficiency versus pillar width ($h_{x}$) for different maximum pillar heights ($h_{y}$) in a ${\mathrm{SiO}}_{2}$-based design ($S_{x}=5$ μm). (**b**) Dependence of efficiency on refractive index (*n*) and pillar height ($h_{y}$); higher-index materials achieve full phase modulation at smaller heights but suffer from increased reflection losses. The red dotted rectangle represents the same data points from (**a**). (**c**) Effect of unit cell size ($S_{x}$) for fixed pillar count, showing reduced efficiency with increasing. (**d**) Efficiency versus $S_{x}$ when $h_{x}=100\;\mathrm{nm}$ and $d_{y}$ scale proportionally, illustrating that larger design spaces require higher population size (*N*) and generation count (*G*) for optimal results.

Figure 4 illustrates the influence of various design parameters on the optical efficiency of the color router. For the results shown in Figure 4a,b, the unit cell size was fixed at $S_{x}=5$ μm.

In Figure 4a, the effects of varying the maximum pillar height ($h_{y}$) and pillar width ($h_{x}$) are analyzed for a silica (${\mathrm{SiO}}_{2}$)-based design. For ${\mathrm{SiO}}_{2}$ with refractive index n=1.45, the pillar height required to achieve a full 2π phase modulation can be estimated as h=λ/(n−1)=1.44 μm at a wavelength of λ=650nm. Accordingly, a design with $h_{y}=1.5$ μm provides complete phase coverage. The parametric trends indicate that reducing $h_{y}$ below this threshold decreases the optical efficiency, as the structure can no longer provide sufficient phase modulation. Conversely, increasing the pillar width $h_{x}$ tends to degrade efficiency, since a larger lateral size limits the device’s ability to precisely control the spatial distribution of transmitted light. A smaller $h_{x}$ increases the number of tunable parameters in the system, allowing the optimization algorithm to explore a richer design space and identify more optimal configurations.

Figure 4b examines the impact of using different dielectric materials while keeping the pillar width constant at $h_{x}=100\;\mathrm{nm}$. Here, the performance of color routers made from ${\mathrm{Si}}_{3}N_{4}$ (n=2) and ${\mathrm{TiO}}_{2}$ (n=2.4) is compared with that of ${\mathrm{SiO}}_{2}$ (n=1.45). Materials with higher refractive indices allow the same 2π phase modulation to be achieved with significantly shorter pillar heights, thus reducing the height-to-width aspect ratio. For instance, at λ=650nm, the required heights for full phase modulation are approximately $h_{y}=650\;\mathrm{nm}$ for ${\mathrm{Si}}_{3}N_{4}$ and $h_{y}=464\;\mathrm{nm}$ for ${\mathrm{TiO}}_{2}$. The colormap in Figure 4b further indicates that increasing $h_{y}$ far beyond these values yields no meaningful improvement in efficiency, as seen for $h_{y}=1.5$ μm in ${\mathrm{Si}}_{3}N_{4}$ and for $h_{y}=800\;\mathrm{nm}$ or 1.5μm in ${\mathrm{TiO}}_{2}$. If preferred, the inverse design could also be implemented for nanoimprint resist as the metasuface material, since it can have similar refractive index as ${\mathrm{SiO}}_{2}$.

An additional observation from Figure 4b is that structures with higher refractive index materials generally exhibit lower optical efficiency. This effect can be attributed to enhanced reflection losses at the air-dielectric interfaces. During forward light propagation, the transmitted field encounters two interfaces: air to the top surface and the bottom surface to air. While the first interface experiences normal incidence, the second interface effectively presents an oblique incidence due to the surface topology of the structure (Figure 2a). A higher refractive index contrast increases Fresnel reflection at both interfaces, thereby reducing the overall transmitted optical power and consequently lowering the optical efficiency. However, this can easily be mitigated with the use of an anti-reflection coating [40].

Figure 4c,d present the effect of unit cell size ($S_{x}$) on the color-routing performance of the nano-patterned surface. These analyses provide insight into how the geometrical scaling of the device impacts the achievable optical efficiency and optimization complexity.

In Figure 4c, the number of pillars was fixed at 50 across the unit cell, while the distance between the surface and the monitor ($d_{y}$) was kept constant for all values of $S_{x}$. The results show that increasing the unit cell size leads to a gradual reduction in optical efficiency when the pillar count remains unchanged. This behavior can be attributed to the reduction in the system’s effective numerical aperture (NA). Similar to the case of metalenses, a larger NA requires a denser spatial sampling of the optical phase to maintain precise control over the transmitted light [41,42]. When the unit cell expands without an increase in the number of pillars, the spatial sampling becomes coarser, limiting the device’s ability to achieve fine phase modulation across the aperture. As a result, the efficiency decreases with increasing $S_{x}$.

This trend is further validated by comparing designs with different bottom surface to monitor distances. Specifically, simulations performed for $d_{y}=1.5$ μm and $d_{y}=3.5$ μm demonstrate that a larger propagation distance ($d_{y}=3.5$ μm) corresponds to a smaller effective NA and consequently yields higher overall efficiency. This confirms that the observed reduction in efficiency for larger unit cell sizes is primarily driven by the interplay between NA and pillar density.

In contrast, Figure 4d investigates cases where the pillar width ($h_{x}$) was fixed at 100 nm, and the surface-to-monitor distance ($d_{y}$) was scaled proportionally with the unit cell size $S_{x}$, thereby maintaining approximately the same NA for all configurations. Under these conditions, increasing $S_{x}$ results in a proportional increase in the number of design parameters, since the total number of pillars grows with the physical width of the unit cell. This expansion of the design space offers greater flexibility but also increases the computational complexity of the inverse design process. To effectively explore this enlarged parameter space and converge to an optimal configuration, both the population size (*N*) and the number of generations (*G*) in the Genetic Algorithm must be increased. As indicated in Figure 4d, higher values of *N* and *G* lead to progressively improved optimization outcomes, underscoring the importance of balancing design dimensionality and computational effort in large-scale metasurface optimization.

A detailed discussion of convergence plots and the associated computational costs for large-scale designs is provided in Supplementary Section S4. It is important to note that computational cost scales primarily with the total simulation domain size rather than the dimensions of the individual unit cell. For example, the computational requirements for simulating three 5 μm unit cells are nearly identical to those for a single 15 μm unit cell. Furthermore, recent advancements in scalable nanoimprint lithography [43] have demonstrated the feasibility of fabricating nano-patterned surfaces over significantly larger areas, which supports the scalability of the design parameters utilized in this work.

### 3.3. Color Router-Based Reflective Display

**Figure 5:**
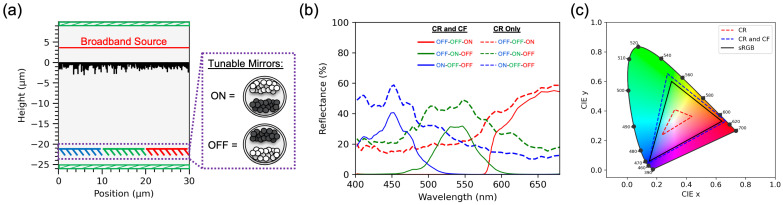
Nano-patterned color router designed for reflective display applications. (**a**) Schematic of the simulation setup showing the color router illuminated by a broadband source and coupled to tunable mirrors that emulate microcapsules in reflective displays. The mirrors switch between reflective (ON) and absorptive (OFF) states to reproduce white and black pixel conditions. (**b**) Simulated reflectance spectra for three color channels under different mirror configurations for the color router (CR) alone and the hybrid color router–color filter (CR–CF) system. The CR–CF configuration exhibits slightly lower reflectance due to additional filter losses. (**c**) Calculated CIE 1931 chromaticity diagram comparing the achievable color gamut of the CR-only and CR–CF systems with the standard sRGB gamut, showing that the hybrid design surpasses the sRGB color space.

We further applied our inverse design methodology to develop nano-patterned color routers tailored for reflective display applications, as shown in Figure 5. The same Genetic Algorithm (GA)-based framework and simulation workflow described in the previous sections were employed for this design. The main modification for this case lies in the geometric scaling of the color router unit cell, which was enlarged to accommodate the dimensions of typical microcapsules used in electrophoretic or electrochromic reflective displays [44,45]. A larger unit cell is therefore essential to ensure proper optical routing across the full aperture of each pixel element in a reflective display configuration.

To validate the color-routing functionality in the reflective display context, we implemented tunable mirrors at the locations corresponding to the virtual monitor planes used during the design phase (Figure 5a). These mirrors emulate the behavior of the microcapsules that can switch between a reflective (white) state and an absorptive (black) state depending on the electrical control signal received from the thin-film transistor (TFT) backplane. The reflectance spectra presented in Figure 5b were obtained through finite-difference time-domain (FDTD) simulations. In each simulated case, one mirror was set to the reflective state while the remaining two were absorptive, thereby isolating the reflection corresponding to a single color channel.

For comparison, we also analyzed a hybrid configuration combining the color router (CR) with conventional color filters (CFs), denoted as the CR–CF hybrid system. The CR-only design achieved an optical efficiency of 55.5%, whereas the CR–CF hybrid exhibited a slightly lower efficiency of 42.5% due to additional absorption and transmission losses introduced by the color filters. However, despite the reduced efficiency, the hybrid configuration produced a significantly wider color gamut, as illustrated in Figure 5c. The resulting gamut surpasses the sRGB standard, demonstrating that color routing can be effectively integrated with conventional filtering approaches to enhance the color reproduction range in reflective display systems.

Overall, these results confirm the adaptability of the proposed inverse design framework across different device architectures, from transmissive CMOS image sensors to reflective display modules, while maintaining efficient color separation and strong wavelength selectivity.

### 3.4. Comparison with Prior Work

Table 1 compares the proposed design with recent state-of-the-art inverse-designed color routers. The optical efficiency is evaluated as the mean of the peak efficiencies of the red, green, and blue channels, ensuring a fair comparison across designs. Prior works employing multilayer configurations demonstrate very high mean optical efficiencies, approaching 99%, owing to the increased degrees of freedom available for phase and amplitude control. However, such multilayer architectures typically involve complex fabrication processes, including multiple deposition and alignment steps, which reduce fabrication compatibility and increase susceptibility to layer-to-layer misalignment. In contrast, single-layer structure implementations offer significantly improved fabrication simplicity and robustness, albeit often at the cost of reduced optical efficiency. Among reported single-layer designs, the present work achieves the highest mean optical efficiency of 75.5%. Furthermore, gradient-based optimization can be sensitive to initialization and prone to convergence to local minima. Gradient-free approaches such as genetic algorithms mitigate this limitation by exploring a broader design space, contributing to improved performance in single-layer configurations.

## 4. Conclusions

In this work, we demonstrated a comprehensive inverse design framework for the development of a manufacturable dielectric nano-patterned surface capable of efficient color routing across both transmissive and reflective optical platforms. Using a Genetic Algorithm (GA)-based optimization approach integrated with full-wave FDTD simulations, we systematically explored the multidimensional design space of height, width, and material parameters to achieve high-efficiency color separation with minimal crosstalk. The GA framework effectively overcame the limitations of gradient-based and heuristic forward-design techniques, consistently converging toward non-intuitive yet superior photonic structures.

For CMOS image sensor-scale implementations, the proposed color router achieved an average optical efficiency of approximately 75.5%, with peak efficiencies of 76%, 72%, and 78% for the blue, green, and red channels, respectively. The optimized structure demonstrated clear wavelength-dependent field localization, validating its ability to spatially route different color components to designated channels without the absorption losses inherent to conventional color filters. We further examined a hybrid configuration combining color routers (CRs) and color filters (CFs), revealing that although the hybrid approach slightly reduced efficiency due to filter absorption, it significantly expanded the achievable color gamut beyond the sRGB standard, offering a viable balance between color accuracy and luminance. An experimental validation pathway of our work has been discussed in Section S5 of the Supplementary Document.

A comprehensive parametric analysis highlighted key design trade-offs. Reduced pillar height compromised phase modulation, while excessive pillar width degraded spatial control and overall efficiency. Additionally, higher-refractive-index materials enabled compact designs but introduced reflection losses that limit throughput. Scaling studies confirmed that optical performance is closely linked to the numerical aperture and spatial sampling density of the structure, establishing practical guidelines for extending the design to larger unit cells.

Finally, the methodology was extended to reflective display configurations. By coupling the color router to tunable mirrors mimicking electrophoretic microcapsules, we demonstrated efficient wavelength-selective reflection and robust color routing suitable for next-generation reflective displays. The proposed CR–CF hybrid design further enabled color reproduction surpassing sRGB while maintaining practical fabrication compatibility.

Overall, the presented GA-based inverse design approach provides a versatile and manufacturable route to engineer multifunctional nano-patterned optical structures. The methodology bridges the gap between high-performance optical routing and scalable device integration, opening pathways toward low-power, high-efficiency imaging and display technologies.

## Figures and Tables

**Table 1:** Comparison with recent state-of-the-art inverse-designed color routers.

Reference	Material	Configuration	Optimization Method	Mean Optical Efficiency
[46]	${\mathrm{SiO}}_{2},{\mathrm{Si}}_{3}N_{4}$	Multi-Layer	Gradient-based	87.2%
[26]	${\mathrm{SiO}}_{2},{\mathrm{TiO}}_{2}$	Multi-Layer	Gradient-based	99%
[27]	${\mathrm{SiO}}_{2},\mathrm{SiN}$	Multi-Layer	Gradient-based	99%
[29]	${\mathrm{Si}}_{3}N_{4}$	Single Layer	Genetic Algorithm (GA)	55.33%
[28]	SiN	Single Layer	Gradient-based	60.67%
[30]	${\mathrm{Nb}}_{2}O_{5}$	Single Layer	Genetic Algorithm (GA)	37.67%
[47]	${\mathrm{TiO}}_{2}$	Single Layer	NSGA-II	51%
This work	${\mathrm{SiO}}_{2}$	Single Layer	Genetic Algorithm (GA)	75.5%

## Data Availability

The original contributions presented in this study are included in the article/Supplementary Material. Further inquiries can be directed to the corresponding author.

## Supplementary Materials

The following supporting information can be downloaded at: https://www.mdpi.com/article/10.3390/nano16040251/s1, Figure S1: Impact of fabrication errors on color router efficiency; Figure S2: Sensitivity to polarization and oblique incidence; Figure S3: Phase distribution profiles of the color router; Figure S4: Considerations for large-scale design using Genetic Algorithm; Figure S5: Experimental validation pathway.

## Abbreviations

The following abbreviations are used in this manuscript:

GA	Genetic Algorithm
CF	Color Filter
CR	Color Router
FDTD	Finite-difference time-domain
