# Supplementary material for: Inverse Design of Optical Color Routers with Improved Fabrication Compatibility
Source: Nanomaterials (Basel). 2026 Feb 14;16(4):251. doi: 10.3390/nano16040251 (PMC12943444; doi:10.3390/nano16040251)
Supplement: Supplementary file 1 [file nanomaterials-16-00251-s001.zip › nanomaterials-4081361-supplementary.pdf]

Supplementary Document for

Inverse Design of Optical Color Routers with Improved Fabrication Compatibility

Sushmit Hossain, Zerui Liu, Nishat Tasnim Hiramony, Tinghao Hsu, Himaddri Roy, Hongming Zhang and Wei Wu*

Ming Hsieh Department of Electrical and Computer Engineering, University of Southern California, Los Angeles, CA 90089, USA

*Corresponding author. Email: wu.w@usc.edu

This document includes:

Figure S1. Impact of fabrication errors on color router efficiency. (a) Sensitivity to uniform height deviations across all nanopillars, showing a peak at the design height (0 nm shift). (b) Sensitivity to random height fluctuations (surface roughness), where efficiency decreases as the maximum range of randomness increases.

Figure S2. Sensitivity to polarization and oblique incidence. (a)Sensitivity analysis of different polarization (b) Sensitivity analysis of oblique incidence.

Figure S3. Phase distribution of the whole simulation space for (a) 450nm, (b)550nm and (c) 650nm wavelength. Phase distribution at the monitor plane for (d) 450nm, (e)550nm and (f) 650nm wavelength.

Figure S4. Considerations for large-scale design using Genetic Algorithm. (a) Convergence plot for different sizes of unit cells. (b) Computational cost for different unit cell sizes, normalized to the 5µm case. The computational cost includes the number of Yee grid cells in simulation, population size and total number of generations.

Figure S5. Experimental validation pathway to compare numerically obtained results.

S1. Fabrication Tolerance of the Color Router

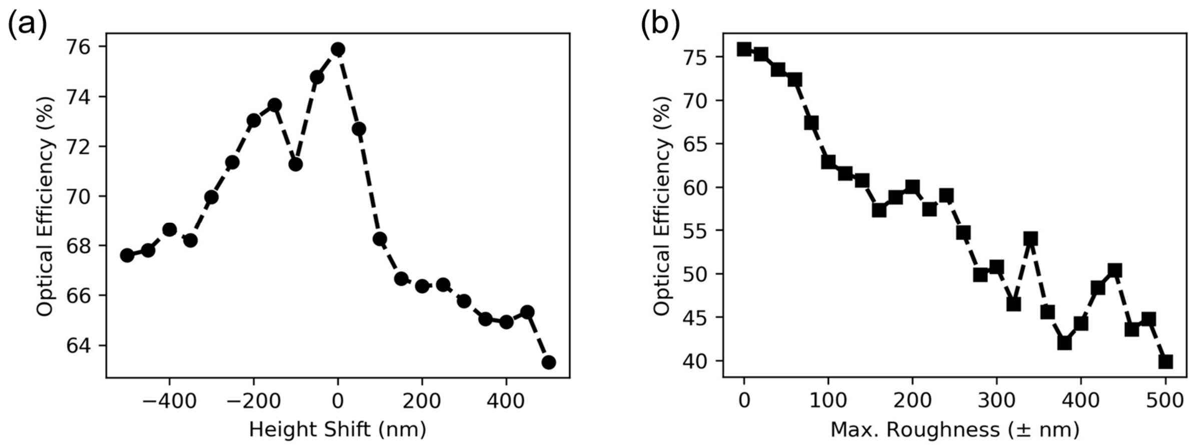

Figure S1. *Impact of fabrication errors on color router efficiency. (a) Sensitivity to uniform height deviations across all nanopillars, showing a peak at the design height (0 nm shift). (b) Sensitivity to random height fluctuations (surface roughness), where efficiency decreases as the maximum range of randomness increases.*

To model realistic fabrication constraints, we performed a sensitivity analysis on the nanopillar geometry. Systematic errors were simulated by applying a global height offset ΔH ranging from -500 nm to +500 nm. The results in Figure S1a show very high optical efficiency even after introducing a height offset. This phenomenon could be attributed to the constant relative phase of the structure-induced field distribution. In parallel, the effect of surface roughness or etching non-uniformity was modeled by assigning each pillar a height, $H_y + \delta$, where δ is a random variable for each pillar within a maximum range. The results in Figure S1b indicate that the color router maintains >60% efficiency within a ± 100 nm tolerance window, but is more susceptible to higher roughness values, which likely induce scattering losses and phase decoherence. Since the realization of continuously varying nanopillar heights can introduce additional challenges in mold fabrication and nanoimprint fidelity, the presented tolerance simulations are intended to evaluate the robustness of the optical response to realistic process-induced variations.

S2. Sensitivity to Polarization and Oblique Incidence

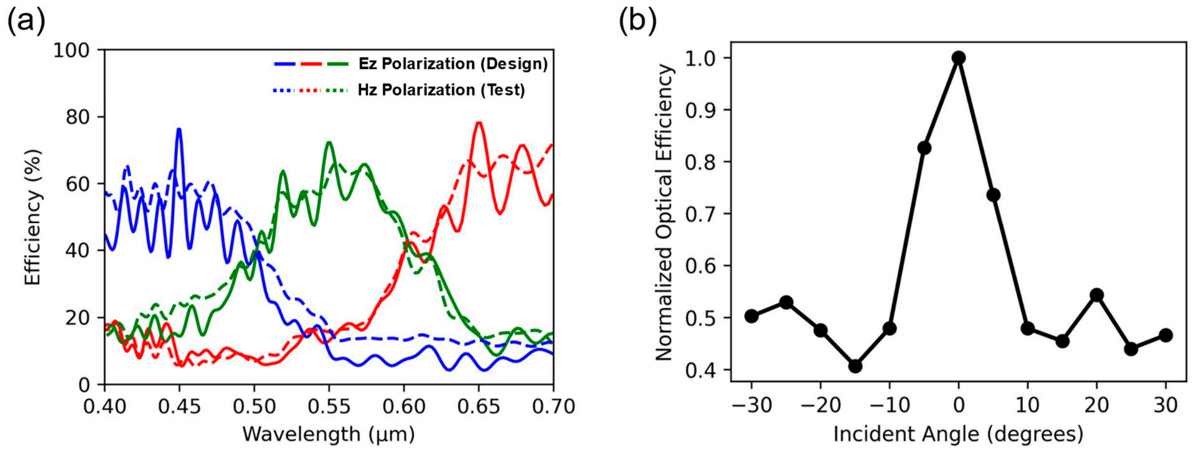

Figure S2. *Sensitivity to polarization and oblique incidence. (a)Sensitivity analysis of different polarization (b) Sensitivity analysis of oblique incidence.*

The height-varying structure serves as a robust color-routing platform that maintains high performance across varying polarization states and incident angles, as detailed in Figure S2. Designed primarily for Ez polarization, where it achieves a peak optical efficiency of 75.5%, the device demonstrates significant polarization insensitivity by retaining around 67% efficiency when tested under Hz polarization. As shown in the spectral analysis of Figure S2a, the routing efficiency remains consistent across the visible spectrum for both polarization states, indicating that the underlying phase-shifting mechanism is broadly applicable. Furthermore, the sensitivity analysis to oblique incidence in Figure S2b reveals an angular tolerance with a Full-Width at Half-Maximum (FWHM) of $\pm$ 10 degrees. This angular bandwidth is sufficient for integration with an F/2.8 imaging lens [1], providing a practical balance between efficiency and system compatibility. While the current single-layer design meets these standard imaging requirements, the range is still lower compared to multi-layer counterparts. Thus, the angular response can be further refined and optimized by incorporating additional layers on top to enhance the device's overall spectral and spatial control.

S3. Phase Distribution and Underlying Physical Mechanism

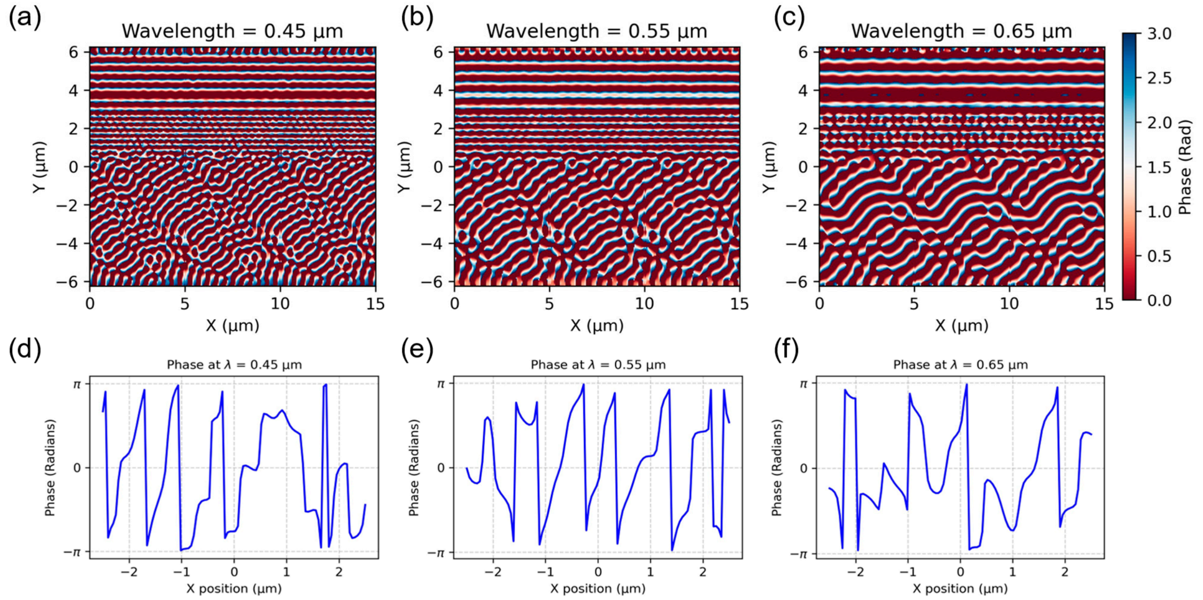

Figure S3. *Phase distribution of the whole simulation space for (a) 450nm, (b)550nm and (c) 650nm wavelength. Phase distribution at the monitor plane for (d) 450nm, (e)550nm and (f) 650nm wavelength.*

Figure S3 illustrates the wavelength-dependent spatial phase response of the optimized color-routing structure at three representative visible wavelengths (λ = 0.45, 0.55, and 0.65 μm). The two-dimensional phase maps show different phase morphologies across wavelengths, indicating strongly dispersive wavefront control rather than achromatic behavior. In particular, the spatial phase gradients vary with wavelength, leading to distinct transverse momentum components (1) imparted to each spectral band.

$$\text{Local phase gradient, } \frac{d\phi}{dx} \propto k_x \quad \ldots\ldots\ldots\ldots\ldots\ldots\ldots\ldots\ldots\ldots(1)$$

One-dimensional phase profiles extracted along the x-direction further confirm that both the magnitude and spatial distribution of the phase gradients depend on wavelength, with nonlinear phase variations and multiple phase discontinuities. According to generalized Snell's law (2), these wavelength-dependent phase gradients result in different refraction or diffraction angles for each wavelength, enabling spatial color separation.

$$sin\theta(\lambda) = \frac{\lambda}{2\pi n}\left(\frac{d\phi(x,\lambda)}{dx}\right) \quad \ldots\ldots\ldots\ldots\ldots\ldots\ldots\ldots\ldots\ldots\ldots\ldots(2)$$

The complex phase patterns observed in the near field suggest that color routing arises from wavelength-selective interference and coupling between subwavelength features and propagating modes, a regime that cannot be captured by simple grating models and motivates inverse electromagnetic design approaches.

S4. Considerations for Large-scale Design using Genetic Algorithm

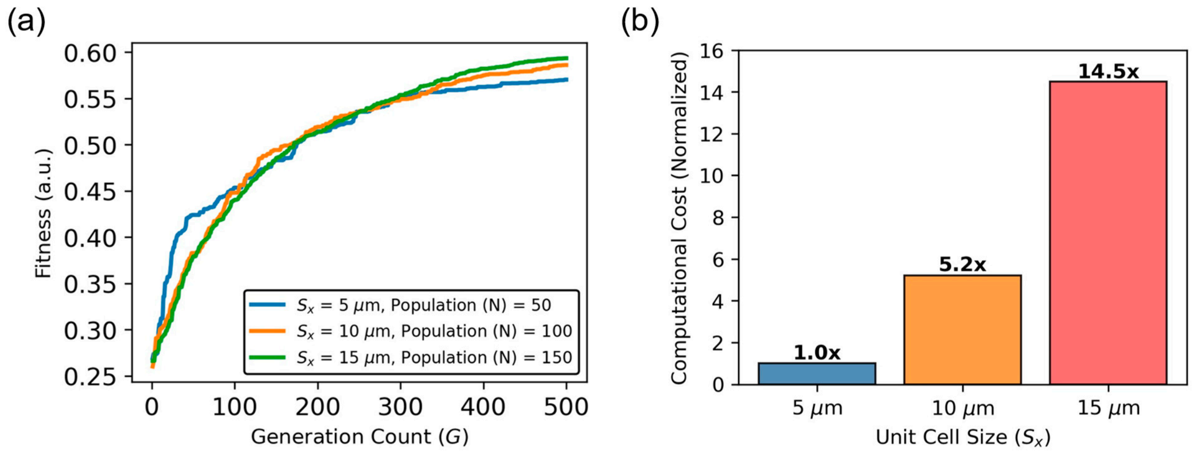

Figure S4. *Considerations for large-scale design using Genetic Algorithm. (a) Convergence plot for different sizes of unit cells. (b) Computational cost for different unit cell sizes, normalized to the 5μm case. The computational cost includes the number of Yee grid cells in simulation, population size and total number of generations.*

To analyze the convergence behavior and computational scalability of our inverse design methodology, we performed a series of optimizations across varying unit cell dimensions, as shown in Figure S4. In Figure S4a, the convergence plots for three distinct unit cell sizes (S_x = 5, 10 and 15μm) are presented. To maintain an effective search of the design space as the number of pillars increases with the unit cell area, we proportionally adjusted the population size (N).

Despite the significant increase in the complexity of the design space for larger unit cells, the convergence curves exhibit a remarkably similar trajectory. In all cases, the Genetic Algorithm reaches a stable fitness plateau within a comparable number of generations, suggesting that our optimization framework is robust and that the selected generation count is sufficient to achieve high-performance designs across different scales.

Figure S4b details the corresponding computational cost for these three implementation cases. We define the computational cost as the product of the number of Yee grid cells, the population size (N), and the total generation size(G), $Cost = No. of\ Yee\ Grid\ Cells \times N \times G$ and we normalize it with our baseline case (S_x = 5μm). As expected, the total resource requirement scales nonlinearly with the unit cell size due to the increased volume of the FDTD simulation domain and the larger population sizes required for convergence. This quantitative breakdown illustrates the scalability of our GA-FDTD framework, demonstrating that while the computational investment increases for larger pixels, it remains within a manageable regime for high-performance computing environments.

S5. Experimental Validation Pathway

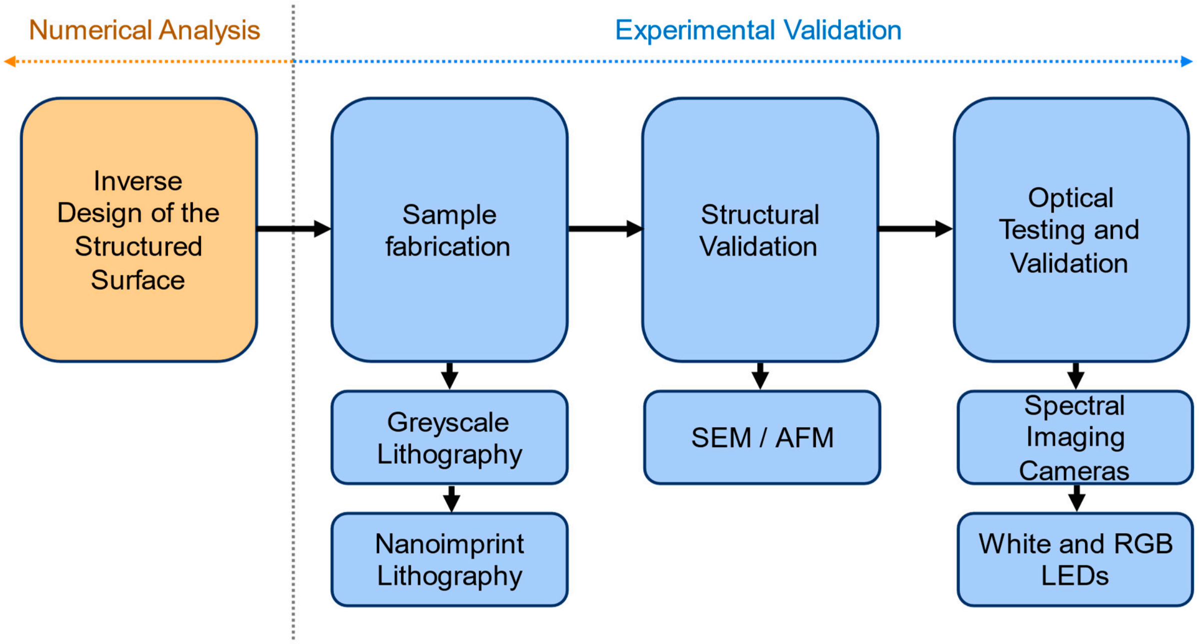

Figure S5. *Experimental validation pathway to compare numerically obtained results.*

To experimentally implement the proposed inverse-designed structures, a feasible validation pathway (as shown in Figure S5) would begin with sample fabrication. Greyscale lithography can be used to realize the multilevel surface profiles obtained from numerical optimization. The patterned resist may then serve as a mother mold, accurately encoding the designed height variations required for wavelength-dependent phase control. This master mold can subsequently be employed in nanoimprint lithography (NIL) to enable scalable replication. In the NIL process, the mold would be pressed into a UV-curable or thermoplastic resist layer deposited on the target substrate, followed by curing and mold release to transfer the nanostructured pattern. The resist layer with a refractive index very close to SiO_2 can be chosen for this task. For any other refractive index implementation, the optimization would need to be redone to avoid any experimental mismatch. The NIL approach allows high-throughput fabrication while maintaining subwavelength feature fidelity.

Structural validation of the fabricated samples could be performed using scanning electron microscopy (SEM) and atomic force microscopy (AFM). SEM imaging would enable inspection of lateral feature dimensions, pattern uniformity, and defect formation, while AFM measurements could be used to quantify surface topography, height profiles, and roughness for finer structures. Comparisons between measured and designed geometries would provide an assessment of fabrication accuracy.

Optical property validation could be carried out by characterizing the wavelength- and position-dependent optical response of the structure. Previous studies have used white and R/G/B LEDs in conjunction with microscopes and spectral imaging cameras for this task. [2,3] Broadband or narrowband illumination sources, such as white or RGB LEDs and single-wavelength lasers, could be used to illuminate the device. The optical signal at each predefined monitor location could be collected using imaging optics and analyzed with spectral imaging cameras or fiber-coupled spectrometers to obtain local spectra. Single-wavelength intensity distributions could be measured using monochromatic illumination or bandpass filters in combination with calibrated CMOS or CCD cameras. These measurements would enable direct comparison with simulated spectral and spatial intensity responses.

References

[1] Catrysse, P. B., Zhao, N., Jin, W., & Fan, S. (2022). Subwavelength Bayer RGB color routers with perfect optical efficiency. *Nanophotonics*, *11*(10), 2381-2387.

[2] Kim, C., Hong, J., Jang, J., Lee, G. Y., Kim, Y., Jeong, Y., & Lee, B. (2024). Freeform metasurface color router for deep submicron pixel image sensors. *Science Advances*, *10*(22), eadn9000.

[3] Hsu, W. L., Yu, C. Y., Huang, Y. S., Zeng, Q. C., Chen, Y. C., Lu, G. H., ... & Wang, C. M. (2025). Simplest but Efficient Design of a Color Router Optimized by Genetic Algorithms. *Acs Photonics*, *12*(3), 1402-1408.
